# Wernekink Commissure Syndrome With Bilateral Cerebellar Signs and Holmes Tremor in a Patient With a Preexisting Movement Disorder

**DOI:** 10.7759/cureus.35674

**Published:** 2023-03-01

**Authors:** Ketan Agarwal, Ratnadeep Biswas, Vijay Kumar

**Affiliations:** 1 Department of General Medicine, All India Institute of Medical Sciences, Patna, IND

**Keywords:** neurology, neuropathology, ischemic stroke, tegmentum mesencephali, mesencephalon, movement disorders, stroke, rare diseases

## Abstract

Wernekink commissure syndrome (WCS) is an extremely rare midbrain syndrome, in which there is the selective destruction of the decussation of the superior cerebellar peduncle, which commonly presents with bilateral cerebellar signs. We describe a case of WCS with Holmes tremor in a patient having an undiagnosed involuntary movement disorder since childhood following an undocumented case of meningitis. The patient presented with sudden onset gait instability with bilateral cerebellar signs (more prominent on the left side), Holmes tremor in bilateral limbs, slurred speech, and marked dysarthria. No ophthalmoplegia or palatal tremors were noted. The patient was conservatively managed along the lines of a stroke, and there was a marked improvement in cerebellar signs and Holmes tremor with time but no evolution (improvement or worsening) was observed in the involuntary movements of limbs and face that were present before the onset of WCS.

## Introduction

In neurology, focal signs are vital indicators that help clinicians clinch the diagnosis and localize the site of the lesion, and thus, it’s vital to report all different clinical syndromes, especially rare ones. There exists a wide variety of clinical manifestations for midbrain infarcts; some are fairly common but some are rarely encountered. One such rare manifestation is Wernekink commissure syndrome (WCS). Due to the prominent bilateral cerebellar symptoms, the differential for WCS is complicated, and localization and lateralization are quite difficult. We describe a case of a patient with sudden onset of cerebellar symptoms and Holmes tremor, with a history of undiagnosed involuntary choreiform movements since childhood. In this case, due to the presence of symptoms since childhood, the diagnosis becomes particularly challenging. And to the best of our knowledge, such a case of WCS in a patient with a preexisting movement disorder has not been previously reported.

## Case presentation

History and examination

A female patient in her forties, with a history of undiagnosed involuntary movements, presented with a sudden onset of gait instability, inability to stand with swaying to the left side, inability to put on slippers, and inability to carry out day-to-day activities with her hands due to incoordination, such as grasping food or holding a glass of water and bringing it close to the mouth for 10 days. When the patient was young, aged around two years, there was an episode of undocumented meningitis following which there has been a history of involuntary movements of all four limbs and the face ever since, but the patient was capable of doing her activities of daily living. For the last 10 days, there was a loss of coordination in performing simple activities. On examination of the neurological system, higher mental functions were intact. A noticeable change in the speech was observed - comprehension, naming, and repetition were normal, but the speech was dysarthric with slurred pronunciation. The patient was able to understand and follow commands. All cranial nerves were within normal limits. Motor examination revealed good muscle tone with normal strength of all major muscle groups. Gag and palatal reflexes were present. A jaw jerk was present. Involuntary, irregular choreiform movements of both upper and lower limbs (involving the distal extremities more), also involving the face, were noted at rest. Occasional facial grimacing and *Jack-in-the-Box* tongue were present as well. Sensory examination was unremarkable, with sensations of pain and light touch intact bilaterally. Cerebellar signs (more prominent on the left side), i.e., intention tremors, dysdiadochokinesia, heel-shin test, and swaying to the left were present. Holmes tremor (a low-frequency tremor exaggerated on movement) was seen bilaterally and present at rest and as intention tremors with a postural component as well. Marked dysarthria was noted. No tongue or palatal tremors were seen. Gait was unsteady. No signs of meningeal irritation were seen.

Differential diagnoses

Differential diagnoses, such as bilateral cerebellar infarcts, isolated bilateral middle cerebellar peduncle infarcts, acute viral cerebellitis, and medication-induced cerebellitis must be taken into account when a patient exhibits bilateral cerebellar impairment [1]. But an MRI of the brain helped rule out these differentials along with the normal white blood cell counts of blood and cerebrospinal fluid and a detailed medication history.

Investigations

Complete blood count, liver function test, and kidney function tests were under normal limits. Cerebrospinal fluid analysis was done to rule out any central nervous system (CNS) infections, and the findings were unremarkable. Electrocardiogram showed normal sinus rhythm. Carotid doppler did not show any atherosclerotic changes. For investigating the cause of the choreiform movements since childhood, the Venereal Disease Research Laboratory (VDRL) test and human immunodeficiency virus (HIV) rapid kit test were conducted (results were negative). Serum ceruloplasmin levels, 24-hour urinary copper levels, and antistreptolysin O (ASO) titer were normal. Two-dimensional transthoracic echocardiography results were unremarkable as well. Genetic analysis could not be done due to financial constraints. Due to the lack of any medication intake, no significant family history, and the presence of a history of meningitis before the onset of those involuntary movements, we suspected infectious chorea.

**Figure 1:**
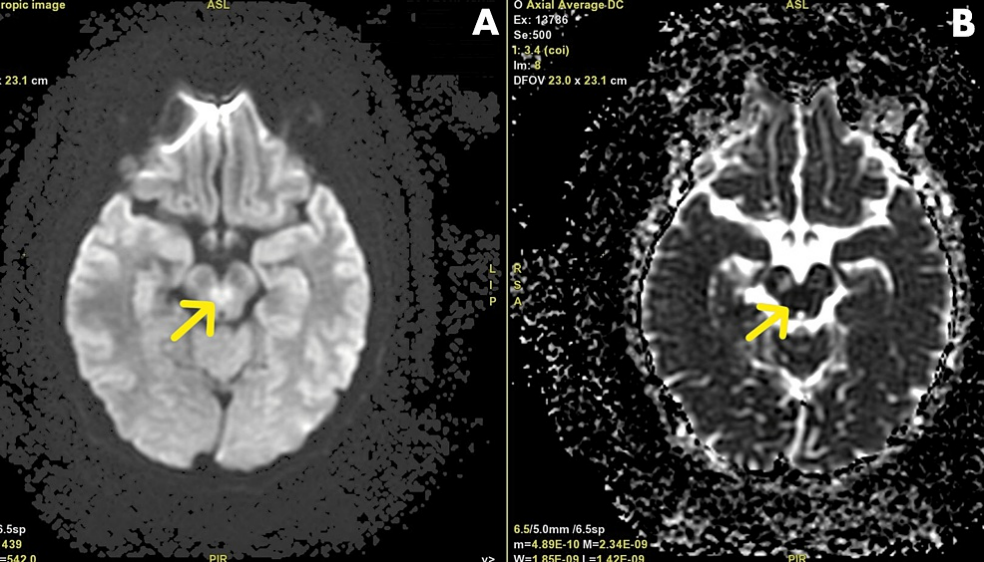
Brain MRI of the patient showing (A) an area of hyperintensity seen in bilateral tegmentum in DWI sequence and (B) appearing hypointense in ADC mapping, suggestive of Wernekink commissure syndrome. The arrows in both panes A and B point to the areas showing the features mentioned in the corresponding panes. MRI, magnetic resonance imaging; DWI, diffusion-weighted imaging; ADC, apparent diffusion coefficient

**Figure 2:**
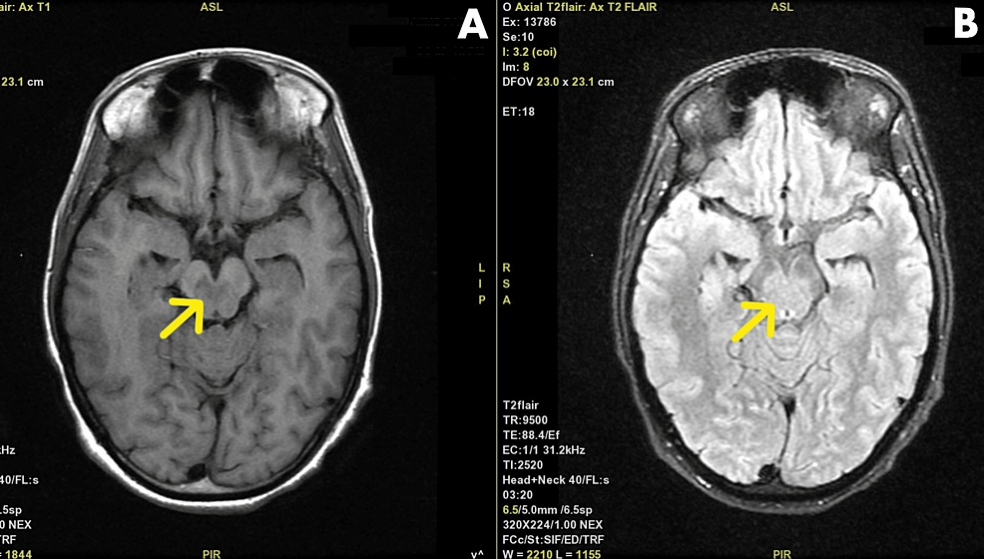
Brain MRI of the patient showing (A) an axial section of T1-weighted image showing hypointensity in bilateral tegmentum and (B) a T2-FLAIR image showing hyperintensity in the corresponding area. The arrows in both panes A and B point to the areas showing the features mentioned in the corresponding panes. MRI, magnetic resonance imaging; T2-FLAIR, T2-weighted fluid-attenuated inversion recovery

The brain MRI confirmed the diagnosis of WCS as it showed diffusion-weighted imaging (DWI) restriction in the tegmentum of the midbrain at the level of inferior colliculi along the interpeduncular fossa (Figures 1-2).

Treatment, outcome, and follow-up

After initial stabilization, conservative management of stroke was initiated, which included hydration, nutritional support, physiotherapy, and occupational therapy. The patient was started on antiplatelets and high-dose statins for secondary prevention. The patient showed significant improvement in coordination and dysarthria during the hospital stay of almost 15 days. After one month, the patient was followed up and showed marked improvement in all cerebellar signs and Holmes tremor. She could now walk independently and was able to do almost all of her daily activities that could before the infarction with a near-complete return to functionality as before. But no appreciable changes were observed in the choreiform involuntary movements of the limbs and face that were present since before the infarction.

## Discussion

The horseshoe-shaped *commissure of Wernekink* was the name given to the decussation of the superior cerebellar peduncle, which lies at the level of the inferior colliculi. It contains the dentothalamic and cerebellorubral tracts and thus connects the cerebellum to the thalamus and red nucleus [2]. WCS is an extremely rare midbrain syndrome, in which there is the selective destruction of this decussation. A few different etiologies have been ascribed to this syndrome such as infarction, hemorrhage, Hashimoto's encephalopathy, neuromyelitis optica spectrum disease, vascular malformation, and demyelinating pseudotumor [3]. Paramedian/central caudal midbrain infarctions have been reported more as the primary etiology among the few cases reports available on WCS [4,5].

Although in this patient, an undiagnosed involuntary movement disorder did exist previously, infarction led to the sudden development of WCS. The presence of involuntary movements of the limbs, in particular, obscured the cerebellar findings at first glance, but in-depth history and neurological examination helped discern the new-onset bilateral cerebellar signs and Holmes tremor. It is worth noting that the patient showed considerable improvement in gait stability, ability to walk, and doing her day-to-day activities during the hospital stay and afterward; yet, the choreiform involuntary movements remained as they were with no noticeable evolution. Thus, it is a logical assumption that the neural pathways involved in both the CNS insults (i.e., the one that led to the onset of the choreiform movements of all limbs and faces since childhood and the one that led to WCS) must have been different without considerable overlap.

The Movement Disorder Society's definition of Holmes tremor as a clinical condition includes the triad of the presence of both resting and intentional tremor with a frequency below 4.5 Hz and onset with a varied lag between the lesion and the onset of symptoms [6]. It has been linked to pathogenic lesions that affect the Guillain-Mollaret triangle or the cerebellothalamocortical tract [7,8]. While it is thought that in WCS the damage to the dentatorubrothalamic or dentatorubroolivary pathway may be the reason behind the occurrence of both palatal myoclonus/tremors and Holmes tremor [9], it was interesting to see that our patient, despite having Homes tremor, did not exhibit palatal signs. Furthermore, it should be highlighted that palatal signs have been reported more frequently associated with WCS than Holmes tremor [5]. This points to the need for further evaluation and studies into the pathophysiology of both Holmes tremor and palatal myoclonus, which may help us identify the anatomical basis of differentiation between these two clinical entities.

Other findings that have been frequently reported in WCS are ophthalmoplegia and nystagmus [3,5,10]. In their study, Zhou et al. reported that in 12 patients with caudal paramedian midbrain infarction, diplopia and internuclear ophthalmoplegia (INO) were frequently seen [11]. But unlike these cases and unlike the findings of Yang et al., who reported the presence of some kind of extraocular muscle dysfunction in all four of their cases, we did not observe any prominent ocular findings [12]. Liu et al. did report that only one out of the two cases of WCS had INO [13]. It has been hypothesized that the cause of these ocular findings, especially INO, is the damage to the medial longitudinal fasciculus (MLF), which lies near the Wernekink commissure [9]. Thus, it is likely that in cases where the infarcts may be isolated to the commissure only and not involve the MLF, the ocular findings may not be present, which could be one explanation for the lack of them in our patient.

WCS is most often treated like other ischemic cerebrovascular accidents, such as with antiplatelet aggregation medications, statins, physiotherapy, occupational therapy, etc. Our patient showed considerable improvement during the hospital stay and upon follow-up after a month - most symptoms of WCS had resolved considerably, resulting in the restoration of much of the quality of life to the preinfarction period. Interestingly, a significant improvement was also noted in Holmes tremor as well without the need for additional medical therapies (such as Levodopa, Clonazepam, Levetiracetam, or Trihexyphenidyl) or therapies such as deep brain stimulation, which have been recommended for the treatment of Holmes tremor [9]. Therefore, the management of WCS should focus on establishing the diagnosis and underlying etiology first, and then treatment should be initiated based on the identified etiology.

## Conclusions

*Commissure of Wernekink* is the name given to the decussation of the superior cerebellar peduncle, and infarctions are the most frequent causes that lead to its destruction. WCS is extremely rare and thus easy to misdiagnose. The presence of bilateral cerebellar signs should raise suspicion for it. Holmes tremor may also be seen in cases of this syndrome. Clinicians should not always expect ocular or palatal signs (such as ophthalmoplegia or palatal tremors/myoclonus), and the absence of these should not be used to preclude a diagnosis of WCS. The syndrome is generally managed based on the underlying etiology.
